# Novel Galactosidase-Beta-1 Variant in Infantile GM1 Gangliosidosis: A Case Report

**DOI:** 10.7759/cureus.102121

**Published:** 2026-01-22

**Authors:** Preeti Srivastava, Abhishek Kumar, Shikhar Deep Jain, Ratan Kumar, Shikha Swaroop, Tapas Sarangi

**Affiliations:** 1 Pediatrics, Tata Main Hospital, Jamshedpur, IND; 2 Pediatrics, Manipal-Tata Medical College, Manipal Academy of Higher Education, Jamshedpur, IND

**Keywords:** gene therapy, glb1, gm1 gangliosidosis, infantile form, novel variant, whole-exome sequencing

## Abstract

GM1 gangliosidosis is an autosomal recessive lysosomal storage disorder caused by pathogenic *GLB1* variants that impair β-galactosidase activity, resulting in GM1 ganglioside accumulation. The infantile (type I) form is the most severe. We describe the case of a one-year-old girl born to consanguineous parents who presented with developmental regression, hypotonia, coarse facial features, hepatosplenomegaly, macular cherry-red spots, Mongolian spots, and sensorineural hearing loss. Whole-exome sequencing revealed a novel homozygous *GLB1* variant, NM_000404.4:c.1525T>A (p.Trp509Arg), absent from population databases and predicted deleterious by in silico tools. According to the American College of Medical Genetics and Genomics guidelines, it is classified as a variant of uncertain significance, though the phenotype-genotype match suggests pathogenicity. This case broadens the *GLB1* mutational spectrum and underscores the value of early genetic testing for diagnosis, counseling, and management.

## Introduction

GM1 stands for monosialotetrahexosylganglioside, a glycosphingolipid containing a sialic acid residue found in neuronal cell membranes, with potential neuroprotective and neuroregenerative activities. Upon administration, GM1 can both prevent neurologic damage and induce regeneration of damaged neurons through neurotrophic repair mechanisms, enhancement of the production of neurotrophins, and augmenting neurite outgrowth. In addition, GM1 exerts anti-excitotoxic activity, prevents necrosis, and improves neuronal recovery and function [1].

GM1 gangliosidosis arises from a deficiency of acid β-galactosidase, encoded by *GLB1* on chromosome 3p21.33 [2,3]. This lysosomal enzyme degrades GM1 ganglioside, a sialic acid-containing glycosphingolipid enriched in neuronal membranes, and other substrates. Pathogenic *GLB1* variants disrupt this process, causing GM1 build-up in neurons and glycolipid storage in visceral organs. The gene also produces a 67 kDa elastin-binding protein involved in extracellular matrix assembly. Variants in *GLB1* underlie both GM1 gangliosidosis and Morquio B disease, with over 130 reported changes including missense, nonsense, and indels [4].

GM1 gangliosidosis has three clinical forms. Type I (infantile) is the most aggressive, emerging before 6-12 months with hypotonia, neurodevelopmental regression, coarse facies (e.g., macroglossia, depressed nasal bridge), hepatosplenomegaly, and a classic macular cherry-red spot on fundoscopy. Additional signs often include Mongolian spots, skeletal changes, cardiomyopathy, seizures, and hearing loss. Life expectancy rarely exceeds three years due to central nervous system decline. Types II (late-infantile/juvenile) and III (adult) progress more slowly with later onset. Definitive diagnosis relies on low β-galactosidase activity and/or biallelic *GLB1* variants. Here, we report infantile GM1 gangliosidosis in a girl with a novel homozygous *GLB1* variant (c.1525T>A, p.Trp509Arg), detailing the clinical features, variant assessment per the American College of Medical Genetics and Genomics (ACMG) criteria, and implications [5].

## Case presentation

**Figure 1:**
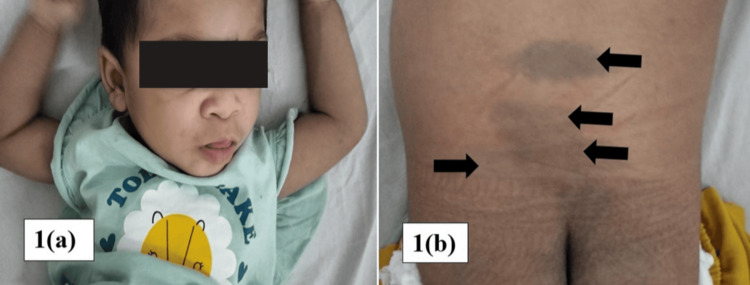
(a) Facial dysmorphism: thick eyebrows, depressed nasal bridge, broad nasal tip, low-set and posteriorly rotated ears, facial puffiness, thick skin, and protruding tongue. (b) Multiple Mongolian spots on the sacrum and buttocks.

A one-year-old female, the first child of healthy consanguineous parents, had an unremarkable antenatal and birth history but presented with global developmental delay and regression. She gained head control at four months but lost it by seven months and never sat unsupported. On examination, there was facial dysmorphism with gingival hypertrophy (Figure 1a). Anthropometry, including head circumference, was in the normal range. Multiple Mongolian spots were visible on the sacrum and buttocks (Figure 1b). She had hepatomegaly and mild splenomegaly, hypotonia, and exaggerated startle reflex.

Brainstem evoked response audiometry was done as a part of the evaluation of the global developmental delay workup, which confirmed bilateral sensorineural hearing loss.

**Figure 2:**
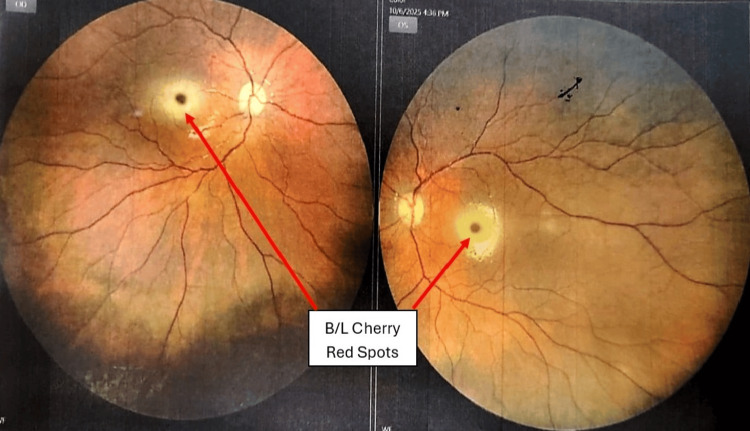
Bilateral cherry-red spots.

Fundoscopic examination revealed bilateral macular cherry-red spots (Figure 2). Contrast-enhanced MRI of the brain and electroencephalogram (EEG) were normal. Routine blood investigations were unremarkable except for mild aspartate aminotransferase (AST) elevation at 141.60 U/L (reference range = 0-35) U/L, and alanine aminotransferase (ALT) was 41.50 U/L (reference range = 0-45) U/L. Given the constellation of developmental regression, visceromegaly, dysmorphic facies, and cherry-red macula, a lysosomal storage disorder was suspected.

Whole-exome sequencing identified a homozygous *GLB1* variant NM_000404.4:c.1525T>A (p.Trp509Arg) (Table 1). This missense change affects a highly conserved residue and is predicted to be deleterious by multiple in silico tools. It is absent in population databases (gnomAD, 1000 Genomes) and not previously reported in ClinVar or other literature, indicating a novelty. No other explanatory variants were identified.

**Table 1:** Whole-exome sequencing demonstrating variant of uncertain significance related to the given phenotype. VS = very strong; S = strong; M = moderate; Sup = supporting

Gene (transcript)	Location	Variant	Zygosity	Disease (OMIM)	Inheritance	Classification
*GLB1* (-) (ENST00000307363.10) NM_000404.4	Exon 15	c.1525T>A (p.Trp509Arg)	Homozygous	GM1-gangliosidosis, type I (OMIM#230500) GM1-gangliosidosis, type II (OMIM#230600) GM1-gangliosidosis, type III (OMIM#230650) Mucopolysaccharidosis type IVB (Morquio) (OMIM#253010)	Autosomal recessive	Uncertain significance (PM2_M, PP3_Sup)

Parental testing confirmed both parents to be heterozygous carriers. Therefore, based on ACMG-AMP guidelines, the variant met PM2 (absent from controls) and PP3 (multiple computational predictions), but lacked segregation or functional evidence, leading to classification as a variant of uncertain significance. Nevertheless, the genotype correlated with the phenotype of GM1 gangliosidosis.

## Discussion

The presentation of the index child, including hypotonia, regression, coarse facies, hepatosplenomegaly, and a macular cherry-red spot with onset in infancy, is typical of type I GM1 gangliosidosis [1,4]. The presence of multiple Mongolian spots further supports this diagnosis, as reported in infantile GM1 cases [2,6,7]. Infantile GM1 often shows biochemical markers such as elevated AST with normal ALT [3], findings consistent with our case.

Molecular analysis confirmed a novel *GLB1* variant, c.1525T>A (p.Trp509Arg), in homozygosity. The tryptophan at position 509 is conserved across species, suggesting functional importance. The substitution to arginine introduces a large, charged residue in place of a bulky aromatic amino acid, likely perturbing enzyme structure or substrate binding. Consistent with this, in silico tools (PolyPhen-2, SIFT, etc.) predict a damaging effect. According to ACMG criteria [5], the absence of this variant from large databases (PM2) and concordant damaging predictions (PP3) support pathogenicity. However, without functional enzyme assays or additional cases, we cannot unequivocally label it as pathogenic. Therefore, it remains a variant of uncertain significance.

Nonetheless, the genotype-phenotype correlation in this case is strong. Over 130 distinct *GLB1* mutations have been reported in GM1 patients, including many missense changes associated with type I disease. Notably, Chang et al. found that novel *GLB1* mutations were identified in isolated ethnic cohorts of GM1 patients [1]. Similarly, Arash-Kaps et al. found seven novel *GLB1* variants in a European cohort and emphasized that genotype-phenotype correlation is usually clear [4].

Therapeutically, options are limited. Supportive management (nutrition, physiotherapy, seizure control) remains the mainstay [3]. Experimental therapies for GM1 gangliosidosis (genetic substrate reduction, chaperones, gene therapy) are under investigation but not yet standard [8-10]. Bone marrow transplantation has been performed for a patient with infantile GM1-gangliosidosis, which helped in normalizing the leucocyte β-galactosidase levels but failed to have an impact on neurological deterioration [11]. Gene therapy for GM1 gangliosidosis uses the principle of replacing the deficient gene with either intravenous administration of an adenoviral vector or intracerebroventricular injection of an adeno-associated virus containing cDNA for β-galactosidase, but there are multiple challenges in translating gene therapy to patients with this condition [10]. Early molecular diagnosis facilitates recurrence risk counseling (25%) and prenatal planning. Reporting this novel variant adds to the global *GLB1* mutation registry, aiding future diagnosis in similar cases.

This single-case report lacks functional validation of the variant (e.g., enzyme assays in patient fibroblasts) and additional unrelated cases for segregation evidence, limiting definitive pathogenicity proof. Normal MRI/EEG early in the disease also reflects the challenge of imaging in nascent GM1, where changes emerge later. Biochemical enzyme activity was not measured due to resource constraints.

## Conclusions

We report a novel homozygous *GLB1* variant (c.1525T>A, p.Trp509Arg) in classical infantile GM1 gangliosidosis, expanding the gene’s mutational landscape. Despite being a variant of uncertain significance, the clinical correlation and rarity support its likely disease-causing role. Prompt whole-exome sequencing proved pivotal for diagnosis in this consanguineous family, emphasizing its role in rare disease workups to enable counseling and targeted care.
